# Uncertainty Quantification and Interpretability for Clinical Trial Approval Prediction

**DOI:** 10.34133/hds.0126

**Published:** 2024-04-15

**Authors:** Yingzhou Lu, Tianyi Chen, Nan Hao, Capucine Van Rechem, Jintai Chen, Tianfan Fu

**Affiliations:** ^1^ School of Medicine, Stanford University, Stanford, CA, USA.; ^2^ Computer Science Department, Rensselaer Polytechnic Institute, Troy, NY, USA.; ^3^ Stony Brook University Hospital, Stony Brook, NY, USA.; ^4^ Computer Science Department, University of Illinois Urbana-Champaign, Urbana, IL, USA.

## Abstract

**Background:** Clinical trial is a crucial step in the development of a new therapy (e.g., medication) and is remarkably expensive and time-consuming. Forecasting the approval of clinical trials accurately would enable us to circumvent trials destined to fail, thereby allowing us to allocate more resources to therapies with better chances. However, existing approval prediction algorithms did not quantify the uncertainty and provide interpretability, limiting their usage in real-world clinical trial management. **Methods:** This paper quantifies uncertainty and improves interpretability in clinical trial approval predictions. We devised a selective classification approach and integrated it with the Hierarchical Interaction Network, the state-of-the-art clinical trial prediction model. Selective classification, encompassing a spectrum of methods for uncertainty quantification, empowers the model to withhold decision-making in the face of samples marked by ambiguity or low confidence. This approach not only amplifies the accuracy of predictions for the instances it chooses to classify but also notably enhances the model’s interpretability. **Results:** Comprehensive experiments demonstrate that incorporating uncertainty markedly enhances the model’s performance. Specifically, the proposed method achieved 32.37%, 21.43%, and 13.27% relative improvement on area under the precision–recall curve over the base model (Hierarchical Interaction Network) in phase I, II, and III trial approval predictions, respectively. For phase III trials, our method reaches 0.9022 area under the precision–recall curve scores. In addition, we show a case study of interpretability that helps domain experts to understand model’s outcome. The code is publicly available at https://github.com/Vincent-1125/Uncertainty-Quantification-on-Clinical-Trial-Outcome-Prediction. **Conclusion:** Our approach not only measures model uncertainty but also greatly improves interpretability and performance for clinical trial approval prediction.

## Introduction

Conducting clinical trials is an essential step in the process of developing new medications [1]. In these trials, the reactions of human subjects to potential treatments (such as individual drug molecules or combinations) are evaluated for specific diseases to assess their safety and effectiveness [2]. As of 2020, the worldwide market for clinical trials was valued at 44.3 billion, with projections to increase to 69.3 billion by 2028 [3]. The financial burden of conducting these trials is substantial, often reaching costs of several hundred million dollars [4]. Moreover, these trials typically span several years, partly due to the meticulous and phased approach. Nevertheless, even with this substantial investment of resources and time, the success probability of these trials is still relatively low [5,6]. Clinical trials can be compromised by various issues, including the drug’s ineffectiveness, safety concerns, and flawed clinical trial design [7]. There has been a surge of studies focusing on how to design better clinical trial mechanisms to enhance clinical trial approval prediction, among which the Hierarchical Interaction Network (HINT) [8] stands out as a notable advancement. HINT has greatly enhanced the probability of clinical trial approval prediction before the trial commences, allowing more resources to be allocated to trials that are more likely to succeed by avoiding inevitable failures. However, even in the face of these advancements, trials may still be predicted even if the confidence is not high for them in some uncertain cases. Fortuitously, historical literature suggests that certain algorithms for uncertainty quantification have opened new opportunities in this field. Meanwhile, the extensive historical data on clinical trials and the comprehensive databases on both successful and unsuccessful drugs pave the way for using machine learning models. This raises a pivotal question: Could we utilize the online database and adopt different strategies based on the degree of certainty, thereby increasing the overall clinical trial approval prediction probability?

Despite the HINT model being the current state-of-the-art method in clinical trial approval prediction, eclipsing other methodologies in several aspects, there remains scope for enhancement, particularly in terms of accuracy and false alarm rate. For example, the application of machine learning in the medical field necessitates not only reliance on model predictions but also a critical assessment of the model’s confidence and timely human intervention. This underscores the vital necessity of not solely depending on the HINT model, as excessive dependence on machine predictions without adequate checks can lead to significant risks. Such risks highlight the importance of integrating uncertainty quantification into these models. Regarding uncertainty quantification, various approaches exist, including Bayesian methods, ensemble techniques, evidential frameworks, Gaussian processes, and conformal prediction.

Among the various methods, conformal prediction stands out because of its simplicity and generality in creating statistically rigorous uncertainty sets for model predictions. A key feature of these sets is their validity in a distribution-free context, offering explicit, nonasymptotic guarantees independent of any distributional or modeling assumptions [9]. Nonetheless, the conventional application of conformal prediction in binary classification scenarios has limitations. Specifically, the resulting prediction lacks practical value when a model predicts both positive and negative outcomes for a sample due to uncertainty. To address this, we propose a shift toward selective classification (SC), wherein the model offers predictions only when it has high confidence; otherwise, it abstains from yielding a prediction. This approach can be applied to any pretrained model, ensuring that the model’s predictions are highly probable and specified by human-defined criteria. This method, however, introduces a trade-off between coverage and accuracy, often characterized by a strong negative correlation. Careful consideration of this balance is crucial in practical applications, especially in the sensitive context of medical predictions.

In this paper, we enhance the HINT for general clinical trial approval prediction tasks. We point out the limitation of the current HINT model and adopt the method of SC to quantify the model uncertainty and improve its prediction performance. We also enhance the interpretability of HINT. Empirical experiments indicate that by applying SC to HINT, the model demonstrates significant elevations in key metrics on phase-level approval prediction. This work paves the way for future explorations into more nuanced models that can handle the complexities of clinical trial data, offering a beacon for forthcoming research in the field. Our findings advocate for the continued development and refinement of models like HINT, emphasizing the need for precision and care in predictive analytics within clinical research. The potential for these advancements can significantly impact patient outcomes and improve the efficiency of trial design.

## Methods

### Formulation

A clinical trial is an organized research effort to evaluate the safety and efficacy of a treatment set aimed at combating a target disease set. This is all guided by a detailed plan known as trial protocol, which is applied to a select group of patients. The trial aims to understand how the treatment performs across various patients, assessing not only its effectiveness but also identifying any potential side effects. Now, we formulate them into more detailed terms for better understanding.

**Definition 1**. *Treatment Set*. Imagine our trial has *K_τ_* drug candidates. These form our treatment set, denoted as *T* = {*τ*_1_, ⋯, *τ_K_τ__*}, where *τ*_1_, ⋯, *τ_K_τ__* are *K_τ_* drug molecules being tested in this trial. Our study concentrates on trials to identify new applications for these drug candidates, while trials focusing on nondrug interventions like surgery or device applications are considered outside the scope of this research.

$$T=\left\{\tau_{1},\cdots ,\tau_{K_{\tau }}\right\},$$ (1)

**Definition 2**. *Target Disease Set*. This refers to the specific diseases the trial is targeting. If our trial is addressing *K_δ_* diseases, then our target disease set is represented by *D* = {*δ*_1_, ⋯, *δ_K_δ__*}, with each *δ_i_* symbolizing the diagnostic code {in this paper, we use ICD10 codes (International Classification of Diseases) [10]} for the *i*th disease.

$$D=\left\{\delta_{1},\cdots ,\delta_{K_{\delta }}\right\},$$ (2)

**Definition 3**. *Trial Protocol*. The trial protocols are the guideline plan of the clinical trial. They are articulated in unstructured natural language and encompass both inclusion (+) and exclusion (−) criteria, which respectively outline the desired and undesirable attributes of potential participants. Imagine a clinical trial for a new medication to treat high blood pressure. Inclusion criteria (+) are the requirements participants need to meet to join the trial. This might include: “adults aged 30 to 65 years” or “diagnosed with high blood pressure”. Similarly, exclusion criteria (−) are the factors that would disqualify someone from participating. This might include “pregnant or breastfeeding women” or “those undergoing treatment for cancer”. By this way, these criteria provide details on various key parameters such as age, gender, medical background, the status of the target disease, and the present health condition.

$$P=\left[\pi_{1}^{+},\ldots ,\pi_{Q}^{+},\pi_{1}^{-},\ldots ,\pi_{R}^{-}\right],\;\pi_{k}^{+/-}\;\text{is}\;a\;\text{criterion}.$$ (3)

*Q* (*R*) is the number of inclusion (exclusion) criteria in the trial. The term $\pi_{k}^{+}$ ($\pi_{k}^{-}$) designates the *k*th inclusion (exclusion) criterion within the trial protocol. Each criterion *π* is a sentence in unstructured natural language.

**Definition 4**. *Clinical Trial Approval*. Clinical trial approval refers to a drug passing a certain phase of a clinical trial, which means that the drug has met specific predefined objectives or endpoints for that phase, demonstrating its safety, efficacy, tolerability, or a combination thereof, depending on the trial’s goals. Each phase of clinical trials has distinct purposes and criteria for success.

**Problem 1**. *Clinical Trial Approval Prediction*. Predicting whether a clinical trial will get approval is like forecasting the outcome of a complex process. Clinical trial approval is represented as a binary label, where 1 means that the trial was a success and 0 means that it was not. The approval of a clinical trial is represented as a binary label *ω* ∈ {0, 1}, where *ω* = 1 signifies a successful trial and 0 signifies a failed one. The estimation of *ω*, represented as $\hat{\omega }$, can be formulated through the function *h_ξ_*, such that $\hat{\omega }=h_{\xi }\left(T,D,P\right)$ , where $\hat{\omega }\in \left[0,1\right]$ denotes the calculated probability of a successful approval. In this context, T, D, and P refer to the treatment set, the target disease set, and the trial protocol, respectively.

#### Significance of clinical trial approval prediction

The clinical trial stands out as the most time-consuming and expensive stage in the drug discovery process. Leveraging machine learning for trial optimization and design holds the potential to significantly accelerate the delivery of life-saving therapeutics to patients. Machine learning tools can play a crucial role in proactively notifying practitioners of potential trial challenges, identifying risks, optimizing safety monitoring protocols, and ensuring participant well-being. In addition, these tools can aid in pinpointing suitable patient populations, optimizing sample sizes, refining inclusion and exclusion criteria, and selecting appropriate endpoints and outcome measures.

### Base model: HINT

**Fig. 1.**
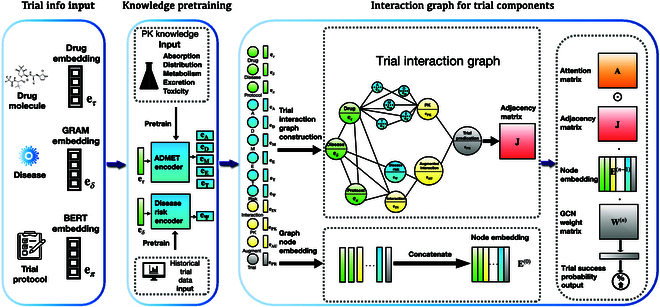
HINT extracts features from the following trial components: drug molecule embedding e*_τ_*, disease embedding e*_δ_*, and trial protocol embedding e*_π_* (as described in Results). Before constructing an interaction graph using these components, HINT pretrains certain embeddings (depicted as blue nodes) using external knowledge about medication characteristics and disease risks (see Discussion). Subsequently, we create an interaction graph in Conclusion to depict the interactions among different trial components. Using this interaction graph, we obtain trial embeddings that represent the trial components and their interactions. Leveraging the learned embeddings and dynamic attentive graph neural network (Eq. 15), we make predictions for trial approvals.

This section describes HINT [8] as the base model. Depicted in Fig. 1, HINT stands as an end-to-end framework, which is innovatively designed to predict the probability of success for a clinical trial before its commencement [8,11]. In the first instance, HINT integrates an input embedding module, where it adeptly encodes multimodal data from various sources, encompassing drug molecules, detailed disease information, and trial protocols into refined input embeddings (see the “Input embedding module” section). Thereafter, these embeddings are fed into the knowledge embedding module to synthesize knowledge embeddings that are pretrained using external knowledge (see the “Pretraining using external knowledge” section). Last, the interaction graph module serves as a nexus, binding these embeddings through an extensive domain knowledge network. This comprehensive interlinking not only unravels the complexity inherent in various trial components but also maps their multifarious interactions and their collective impact on trial approvals. Utilizing this foundation, HINT learns a dynamic attentive graph neural network to prognosticate the trial approval (see the “Hierarchical interaction graph” section).

### Input embedding module

The input of the model contains 3 data sources:

1. Drug molecules play a crucial role in forecasting the approvals of clinical trials. These molecules are typically represented through SMILES (simplified molecular-input line-entry system) strings or molecular graphs [12]. Formally, treatment set *T* = {*τ*_1_, ⋯, *τ_K_τ__*} is represented as

$$\underline{\text{DrugEmbedding}}\;e_{\tau }=\frac{1}{K_{\tau }}\sum_{i=1}^{K_{\tau }}\;g_{\tau }\left(\tau_{i}\right)\in {\mathbb{R}}^{d},$$ (4)

where *g_τ_*(⋅) is designated as the molecule embedding function. By aggregating the molecular embeddings derived from a trial, we obtain drug embedding vector, which is conceptualized as the mean of all molecular embeddings [13]. Our empirical investigations reveal that using an averaging method as the aggregation mechanism for drug embeddings yields more effective results than utilizing a summative approach.

2. Disease information can significantly affect trial approvals. For instance, oncology drugs exhibit lower approval rates compared to those for infectious diseases [14–16]. Disease information is primarily sourced from its descriptive texts and corresponding ontology, such as disease hierarchies like the ICD [10]. Target disease set *D* = {*δ*_1_, ⋯, *δ_k_δ__*} (Definition 2) in the trial can be represented as

$$\underline{\text{DiseaseEmbedding}}\;e_{\delta }=\frac{1}{K_{\delta }}\sum_{j=1}^{K_{\delta }}\;G_{\delta }\left(\delta_{j}\right)\in {\mathbb{R}}^{d},$$ (5)

where *G_δ_*(*δ_j_*) represents an embedding of disease *δ_j_* using GRAM (graph-based attention model) [17], which leverages the hierarchical information inherent to medical ontologies.

3. Trial protocol is a key document that outlines the conduct of a clinical trial and encompasses specific eligibility criteria essential for patient recruitment. These inclusion or exclusion criteria are systematically articulated in individual sentences. To effectively represent each sentence within these criteria, we utilize Clinical-BERT [18]. The derived sentence representations are then sequentially processed through 4 one-dimensional convolutional layers [19], each layer using varying kernel sizes to discern semantic nuances at 4 distinct levels of granularity. This is followed by a fully connected layer that culminates in the formation of the protocol embedding. Concisely, the protocol embedding is characterized as

$$\underline{\text{ProtocolEmbedding}}\;e_{\pi }=g_{\pi }\left(P\right),\;e_{\pi }\in {\mathbb{R}}^{d}.$$ (6)

### Pretraining using external knowledge

HINT integrates external knowledge sources to pretrain knowledge nodes and further refine and augment these input embeddings.

#### Pharmacokinetics Knowledge

We engage in the pretraining of embeddings by harnessing pharmacokinetics (PK) knowledge, which elucidates the body’s reaction to drug absorption. The efficacy of clinical trials is significantly influenced by factors such as the pharmacokinetic properties of a drug and the disease risk profile. In this process, we utilize a spectrum of publicly accessible PK experimental scores. Using this data, our pretraining is directed toward predictive models for key ADMET (absorption, distribution, metabolism, excretion, toxicity) properties. These properties are integral in drug discovery, offering vital insights into the comprehensive interaction of a drug with the human body [15,20]:

1. Absorption model quantifies the period of a drug’s absorption process within the human body.
2. Distribution model evaluates how efficiently the drug molecules traverse the bloodstream and reach various bodily regions.
3. Metabolism model assesses the active duration of the drug’s therapeutic effect.
4. Excretion model gauges the effectiveness of the body in eliminating toxic elements of the drug.
5. Toxicity model appraises the potential adverse effects a drug might have on the human body. For each of these properties, we develop dedicated models to calculate their respective scores and latent embeddings. Our approach involves processing molecular inputs and generating binary outputs, which reflect the presence or absence of the desired ADMET property.

$$\begin{array}{c} \underline{\text{ADMET}}\;\begin{array}{c} e_{*}=\Phi_{*}\left(e_{\tau }\right),\;{\hat{\omega }}_{*}=\sigma \left(\text{FCNN}\left(e_{*}\right)\right)\\ \text{min}\;-\omega_{*}\log{\hat{\omega }}_{*}-\left(1-\omega_{*}\right)\log\left(1-{\hat{\omega }}_{*}\right), \end{array} \end{array}$$ (7)

where **e***_τ_* ∈ ℝ*^d^* is the input drug embedding defined in Eq. 4, *ω*_∗_ ∈ {0, 1} is the binary label, ∗ can be A, D, M, E, and T. FCNN is a one-layer fully connected neural network. *ma*(⋅) represents the sigmoid function that maps the output of FCNN to the binary label *ω*_∗_. Φ_∗_ can be any neural network. Furthermore, we use highway neural network [21], which is denoted as

$$\underline{\text{HighwayNetwork}}\;y=\text{highway}\left(x\right),\;y,x\in {\mathbb{R}}^{d}.$$ (8)

This choice is motivated by the need to mitigate the vanishing gradient problem, a critical consideration in deep neural network training.

#### Disease Risk Embedding and Trial Risk Prediction

Our model extends beyond drug properties, incorporating knowledge gleaned from historical data on trials related to the target diseases. We integrate information from various sources to assess disease risk: (a) disease descriptions and their corresponding ontologies and (b) empirical data on historical trial success rates for each disease. We leverage detailed statistics on the success rates of diseases across different phases of clinical trials, as documented by [16], which serve as a supervision signal for training our trial risk prediction model. More precisely, we utilize previous trial data, available at ClinicalTrials.gov, to predict the likelihood of success for upcoming trials based on the specific disorders involved.

The predicted trial risk, denoted as ${\hat{\omega }}_{\Psi }$, and the embedding, **e**_Ψ_ ∈ ℝ*^d^* are derived using a 2-layer highway neural network (Eq. 8) Ψ(⋅):

$$\begin{array}{c} \underline{\text{DiseaseRisk}}\begin{array}{c} e_{\Psi }=\Psi \left(e_{\delta }\right),\;{\hat{\omega }}_{\Psi }=\sigma \left(\text{FCNN}\left(e_{\Psi }\right)\right),\\ \min-\omega_{\Psi }\text{log}{\hat{\omega }}_{\Psi }-\left(1-\omega_{\Psi }\right)\log\left(1-{\hat{\omega }}_{\Psi }\right), \end{array} \end{array}$$ (9)

where **e***_δ_* ∈ ℝ*^d^* is the input disease embedding in Eq. 5, ${\hat{\omega }}_{\Psi }\in \left[0,1\right]$ is the predicted trial risk between 0 and 1 (with 0 being the most likely to fail and 1 the most likely to succeed), and *ω*_Ψ_ ∈ {0, 1} is the binary label indicating the success or failure of the trial as a function of disease only. FCNN is the one-layer fully connected layer. *σ*(⋅) represents the sigmoid function that maps the output of FCNN to the binary label *ω*_Ψ_. Binary cross-entropy loss between *ω*_Ψ_ and ${\hat{\omega }}_{\Psi }$ is used to guide the training.

### Hierarchical interaction graph

#### Trial Interaction Graph

We have devised a hierarchical interaction graph, denoted as H, which serves as the backbone for establishing connections among all input data sources and the crucial variables that exert influence over the approvals of clinical trials. Below, we provide a comprehensive description of this interaction graph along with its initialization procedure. The interaction graph H is composed of 4 distinct tiers of nodes, each of which is intricately interconnected to reflect the intricate development process of real-world clinical trials. These tiers are as follows:

1. Input nodes encompass drugs, target diseases, and trial protocols with node features of input embedding **e***_τ_*, **e***_δ_*, **e***_π_* ∈ ℝ*^d^*, indicated in green in Fig. 1 (see the “Input embedding module” section).
2. External knowledge nodes include ADMET embeddings **e**_A_, **e**_D_, **e**_M_, **e**_E_, **e**_T_ ∈ ℝ*^d^*, as well as disease risk embedding **e**_Ψ_. These representations are initialized with pretrained external knowledge and are indicated in blue in Fig. 1 (see the “Pretraining using external knowledge” section).
3. Aggregation nodes include (a) interaction node **e**_IN_ connecting disease **e***_δ_*, drug molecules **e***_τ_*, and trial protocols **e***_π_*; (b) PK node **e**_PK_ connecting ADMET embeddings **e**_A_, **e**_D_, **e**_M_, **e**_E_, and **e**_T_, **e***_T_* ∈ ℝ*^d^*; and (c) augmented interaction node **e**_AU_ that augments the interaction node **e**_IN_ using disease risk node **e**_Ψ_. Aggregation nodes are indicated in yellow in Fig. 1.
4. Prediction node: **e**_PR_ node serves as the connection point between the PK node **e**_PK_ and the augmented interaction node **e**_AU_ for making predictions. It is represented in gray in Fig. 1. The input nodes and external knowledge nodes have been previously detailed, and the resulting representations are utilized as node embeddings within the interaction graph. In the following sections, we elaborate on the aggregation nodes and the prediction nodes.

#### Aggregation Nodes

The PK node aggregates information related to the 5 ADMET properties (Eq. 7). We obtain PK embedding as follows:

PKEmbedding¯ePK=PKeA,eD,eM,eE,eT,ePK∈ℝd. (10)

Here, $\mathrm{PK}\left(\cdot \right)$ represents a one-layer fully connected layer (input dimension is 5 × *d*, output dimension is *d*) , whose input feature concatenating **e**_A_, **e**_D_, **e**_M_, **e**_E_, and **e**_T_, followed by *d*-dimensional 2-layer highway neural network (Eq. 8) [21].

Next, we model the interaction among the input drug molecule, diseases, and protocols through an interaction node and obtain its embedding as follows:

InteractionEmbedding¯eIN=ℐNeτ,eδ,eπ,eIN∈ℝd, (11)

where **e***_τ_*, **e***_δ_*, and **e***_π_* represent input embeddings defined in Eqs. 4, 5, and 6, respectively. The neural architecture of $ℐN\left(\cdot \right)$ consists of a one-layer fully connected network (with input dimension is 3 × *d* and output dimension is *d*), followed by a *d*-dimensional 2-layer highway network (Eq. 8) [21].

We also use an augmented interaction model to combine (a) the trial risk associated with the target disease **e**_Ψ_ (Eq. 9) and (b) the interaction among disease, molecule, and protocol represented by **e**_IN_ (Eq. 11).

$$\underline{\text{AugmentedInteraction}}\;e_{\mathrm{AU}}=\mathrm{AU}\left(e_{\Psi },e_{\text{IN}}\right),\;e_{\mathrm{AU}}\in {\mathbb{R}}^{d}.$$ (12)

Here $\mathrm{AU}\left(\cdot \right)$ is a one-layer fully connected network (with input dimension is 2 × *d*, output dimension is *d*), followed by a *d*-dimensional 2-layer highway network (Eq. 8) [21].

#### Prediction Node

Prediction node synthesizes the PK and the augmented interaction to derive the final prediction as follows:

$$\underline{\text{TrialPrediction}}\;e_{\text{PR}}=PR\left(e_{\text{PK}},e_{\text{AU}}\right),\;e_{\mathrm{PR}}\in {\mathbb{R}}^{d}.$$ (13)

Similar to ℐN() and AU(), the architecture of PR consists of a one-layer fully connected network (with input dimension is 2 × *d*, output dimension is *d*), followed by a *d*-dimensional 2-layer highway network (Eq. 8) [21].

### Interpretability: Dynamic attentive graph neural network

Trial embeddings provide initial representations of different trial components and their interactions through a graph. To further enhance predictions, we design a dynamic attentive graph neural network that leverages this interaction graph to model influential trial components.

In mathematical terms, we consider the interaction graph H as the input graph, where nodes represent trial components, and edges denote relations among these components. We denote **J** ∈ {0, 1}^*K*×*K*^ as the adjacency matrix of H. The node embeddings **E**^(0)^ are initialized by combining representations of all components as follows:

$$\begin{array}{l} E^{\left(0\right)}=\left[e_{\delta },e_{\tau },e_{\pi },e_{A},e_{D},e_{M},e_{E},e_{T},e_{\Psi }\right.\\ \;{\left.e_{\text{PK}},e_{\text{IN}},e_{\text{AU}},e_{\text{PR}}\right]}^{⊤}\in {\mathbb{R}}^{K\times d}, \end{array}$$ (14)

*K* =  ∣H∣ is the number of nodes in graph H, and for this paper, *K* = 13.

To enhance node embeddings, we utilize a graph convolutional network (GCN) [22,23]. The updating rule of GCN for the *n*th layer is

$$E^{\left(n\right)}=\text{RELU}\left(B^{\left(n\right)}+\left(A⊙J\right)\left(E^{\left(n-1\right)}W^{\left(n\right)}\right)\right),$$ (15)

where *n* = 1, ⋯, *N*, *N* represents the depth of GCN. In the *n*th layer, **E**^(*n*)^ ∈ ℝ^*K*×*d*^ is node embedding, **B**^(*n*)^, **W**^(*n*)^ ∈ ℝ^*K*×*d*^ are the bias/weight parameter, ⊙ denotes element-wise multiplication.

In contrast to the conventional GCN [22], we introduce a learnable layer-independent attentive matrix $A\in {\mathbb{R}}_{+}^{K\times K}$. **A**_*i*,*j*_, the (*i*, *j*)th entry of **A**, measures the importance of the edge connecting the *i*th and *j*th nodes in H. We calculate **A**_*i*,*j*_ based on the embeddings of *i*th and *j*th nodes in **E**^(0)^, denoted as **e***^i^*, **e***^j^* ∈ ℝ*^d^*, (**e***^i^* ∈ ℝ*^d^* is transpose of the *i*th row of **E**^(0)^ ∈ ℝ^*K*×*d*^ in Eq. 14).

$$A_{i,j}=F\left({\left[{e^{i}}^{⊤},{e^{j}}^{⊤}\right]}^{⊤}\right),\;A_{i,j}>0$$ (16)

where ℱ(·) refers to a 2-layer fully connected neural network with ReLU (rectified linear unit) functions in the hidden layer and sigmoid activation function in the output layer. Notably, the attentive matrix **A** is element-wise multiplied by the adjacency matrix **J** (as shown in Eq. 15) to ensure that edges with higher prediction scores are given more weight during message propagation. Thus, **A**_*i*,*j*_ can be leveraged to conduct interpretability analysis.

#### Training

After the message-passing phase in the GCN, we obtain updated representations for each trial component. These representations encode the essential information learned from the network. To predict trial success $\hat{\omega }$, we feed the final-layer (*N*th layer) representation of the trial prediction node into a one-layer fully connected network with a sigmoid activation function. We use binary cross-entropy loss for training, which measures the dissimilarity between the predicted values and the true ground truth labels.

$$\begin{array}{l} \hat{\omega }=\sigma \left(\text{FCNN}\left(e_{\text{PR}}^{N}\right)\right).\\ L_{\text{classify}}=-\omega \log\hat{\omega }-\left(1-\omega \right)\log\left(1-\hat{\omega }\right). \end{array}$$ (17)

In our case, *ω* ∈ {0, 1} represents the ground truth, with *ω* = 1 indicating a successful trial and 0 indicating a failed one. HINT is trained in an end-to-end manner, optimizing its ability to predict trial approvals based on the learned representations [24]. *σ*(⋅) represents the sigmoid function.

### SC to quantify uncertainty

We consider a binary classification problem, such as clinical trial outcome prediction, which HINT was designed to solve. Let *f* : *X* → *Y* be the model, with feature space *X* (e.g., trial embeddings) and label set *Y* = {0, 1}. Let *P*(*X*, *Y*) be a joint distribution over *X* × *Y*. The selective classifier (*f*, *g*) is made up of the selective function *g* : *X* → {0, 1} and the classifier *f*, which produces a probability for each label provided in the input *x*.

$$\left(f,g\right)\left(x\right)≜\left\{\begin{array}{cc} f\left(x\right), & g\left(x\right)=1;\\ \text{abstain}, & g\left(x\right)=0. \end{array}\right.$$

Therefore, the prediction of input *x* is abstained if *g*(*x*) = 0. We measure the performance of a selective classifier using coverage and risk. Coverage is defined as the probability mass of the nonrejected region of *X*, $\psi \left(f,g\right)=E\left[g\left(X\right)\right]$. Given a loss function L, the selective risk of (*f*, *g*) is defined as

$$R\left(f,g\right)=\frac{E\left[L\left(f\left(x\right),y\right)g\left(x\right)\right]}{\psi \left(f,g\right)}.$$

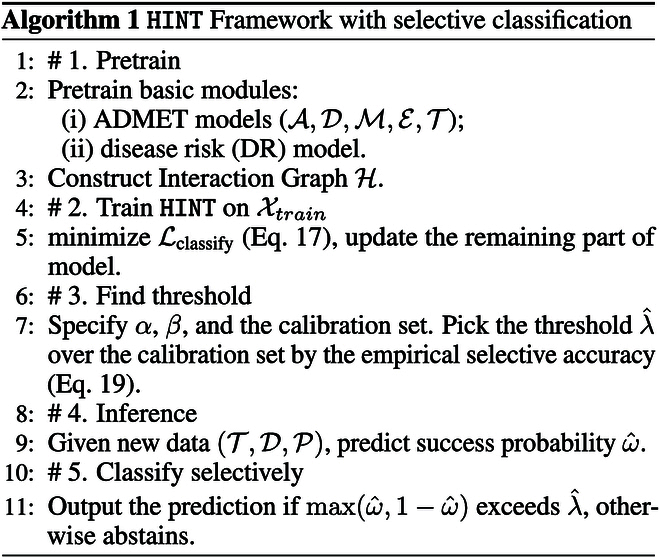


#### Selective Classification

**Fig. 2.**
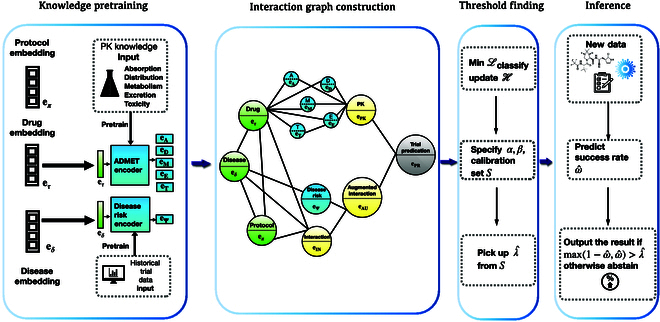
SC on HINT.

In many scenarios, it is preferable to display a model’s predictions only when it has high confidence. For instance, in medical diagnosis, we might only want the model to make predictions if it is 90% certain, and if not, it should say, “I’m uncertain”. The algorithm demonstrated below strategically abstains to achieve higher accuracy in clinical trial outcome prediction tasks, as shown in Fig. 2.

More formally, given sample-label pairs ${\left\{\left(X_{i},Y_{i}\right)\right\}}_{i=1}^{n}$ and a clinical trial outcome predictor $\hat{f}$ , we seek to ensure

$$P\left(Y_{\text{test}}=\hat{Y}\left(X_{\text{test}}\right)|\hat{P}\left(X_{\text{test}}\right)\geq \hat{\lambda }\right)\geq 1-\alpha ,$$ (18)

where $\hat{Y}\left(x\right)={\mathrm{argmax}}_{y}\;\hat{f}{\left(x\right)}_{y}$, $\hat{P}\left(X_{\text{test}}\right)=\mathrm{ma}x_{y}\;\hat{f}{\left(x\right)}_{y}$, and $\hat{\lambda }$ is a threshold calculated upon the calibration set. The accuracy computed over only a subset of high-confidence predictions is called a selective accuracy guarantee. We pick the threshold based on the empirical estimate of selective accuracy on the calibration set.

R^λ=1nλ∑i=1n1Yi≠Y^XiandP^Xi≥λ,wherenλ=∑i=1n1P^Xi≥λ. (19)

where 1(⋅) is an indicator function.

In particular, we will scan across values of *λ*, looking at a conservative upper bound for the true risk (i.e., the top end of a confidence interval for the selective misclassification rate). Realizing that $\hat{R}\left(\lambda \right)$ is a binomial random variable with *n*(*λ*) trials, we upper-bound the misclassification error as

$${\hat{R}}^{+}\left(\lambda \right)=\text{sup}\left\{r:\text{BinomCDF}\left(\hat{R}\left(\lambda \right);n\left(\lambda \right),r\right)\geq \beta \right\}$$ (20)

for some user-specified failure rate *β* ∈ [0, 1]. Then, scan the upper bound until the last time the bound exceeds *α*,

$$\hat{\lambda }=\text{inf}\left\{\lambda :{\hat{R}}^{+}\left(\lambda^{\prime }\right)\leq \alpha \;\text{for}\;\text{all}\;\lambda^{\prime }\geq \lambda \right\}.$$ (21)

Using $\hat{\lambda }$ will satisfy Eq. 18 with high probability.

### Dataset

In our study, we utilized the clinical trial approval prediction benchmark dataset, encompassing 3 phases. We used the TOP clinical trial approval prediction benchmark presented by [8,11]. This dataset encompasses information about drugs, diseases, trial protocol, and trial outcomes for a total of 17,538 clinical experiments. These trials are categorized into 3 phases: phase I with 1,787 trials, phase II with 6,102 trials, and phase III with 4,576 trials. Success rate differs across phases: 56.3% in phase I, 49.8% in phase II, and 67.8% in phase III. A breakdown of the diseases targeted can be found in Table 1. Our research is executed distinctly in each phase of the trials.

**Table 1.** Data statistics of clinical trial approval prediction benchmark dataset. During training, we randomly select 15% training samples for model validation. The earlier trials are used for learning, while the later trials are used for inference. “Succ” and “Fail” are abbreviations for “success” and “failure”, respectively.

Settings	Train		Test		Split date
	Succ	Fail	Succ	Fail	
Phase I	702	386	199	113	13 August 2014
Phase II	956	1,655	302	487	20 March 2014
Phase III	1,820	2,493	457	684	7 April 2014

#### Data Processing and Linking

**Fig. 3.**
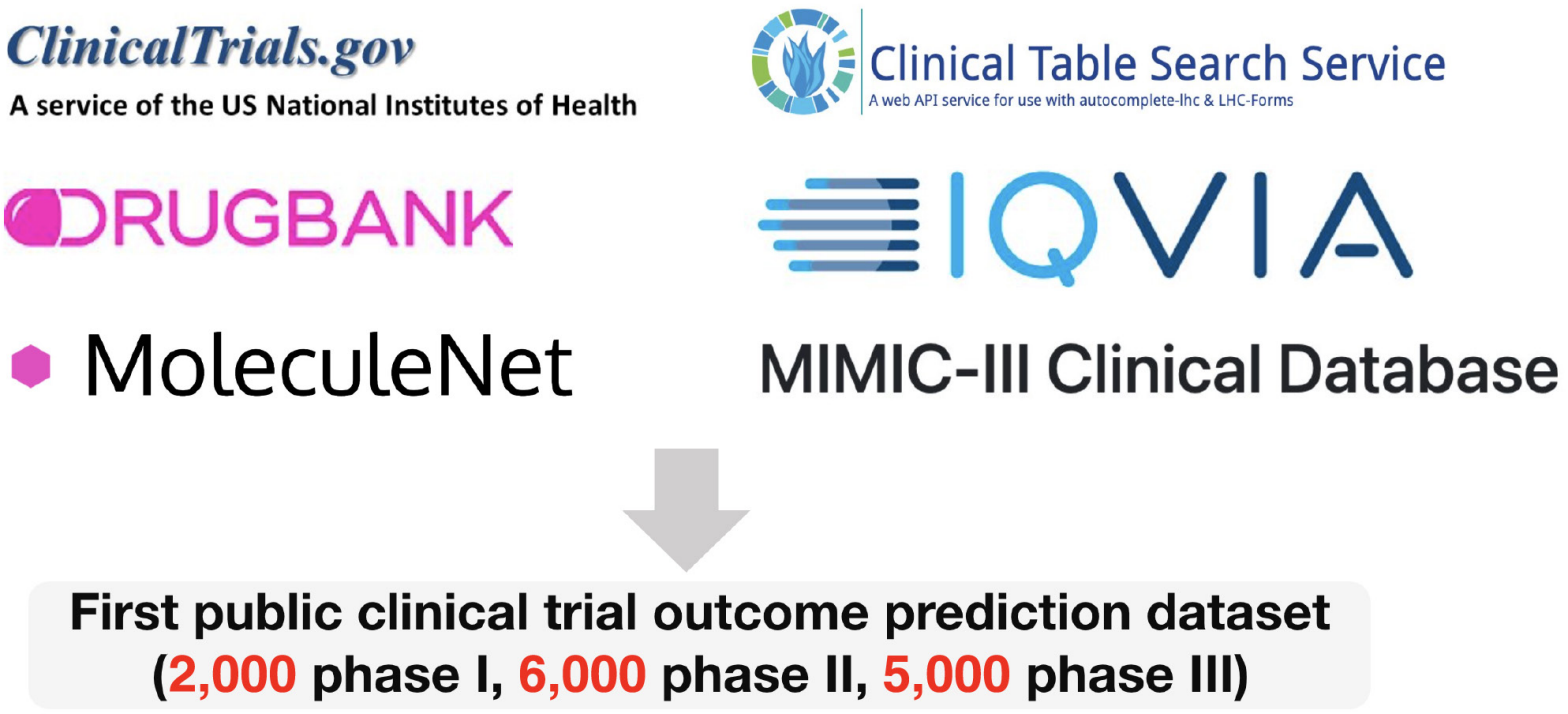
Dataset. The dataset is curated by aggregating multimodal data from various sources. This dataset contains data on medications (drug molecules), diseases, trial protocols (text data), and approval information (labels).

Next, we describe how to process and link the parsed trial data to machine-learning-ready input and output format (Fig. 3):

• Drug molecule data are extracted from https://clinicaltrials.gov/ and linked to its molecule structure (SMILES strings and the molecular graph structures) using DrugBank Database [25] (https://www.drugbank.com/).
• Disease data are extracted from https://clinicaltrials.gov/ and linked to ICD-10 codes and disease description using clinicaltables.nlm.nih.gov and then to CCS (clinical classifications software) codes via hcup-us.ahrq.gov/toolssoftware/ccs10/ccs10.jsp.
• Trial protocol data are extracted from https://clinicaltrials.gov/, in particular, the study description section, outcome section, and eligibility criteria section.
• Trial approval data (binary label) are available at TrialTrove (https://pharmaintelligence.informa.com/products-and-services/data-and-analysis/trialtrove).
• Auxiliary drug PK data include 5 datasets across the main categories of PK. For absorption, we use the bioavailability dataset provided in the supplementary of Ma et al. [26]. For distribution, we use the blood–brain barrier experimental results provided in the study of Adenot and Lahana [27]. For metabolism, we use the CYP2C19 experiment from the paper of Veith et al. [28], which is hosted in the PubChem bioassay portal under AID 1851. For excretion, we use the clearance dataset from the eDrug3D database [29]. For toxicity, we use the ToxCast dataset [30], provided by MoleculeNet (http://moleculenet.ai). We consider drugs that are not toxic across all toxicology assays as not toxic and otherwise toxic. Concretely, we collected all the clinical trial data from https://clinicaltrials.gov/. The historical trial approval probability on each disease (disease risk in our model) is also extracted from this data source. For drug knowledge, the data are extracted from multiple public sources. We obtain drugs’ molecule information from DrugBank Database [25] (https://www.drugbank.com/). For drug property knowledge, we obtain diseases’ ICD-10 code from clinicaltables.nlm.nih.gov.

#### Temporal Data Split based on Start and Completion Date

We leverage temporal split, which refers to splitting the data samples based on their time stamps. The earlier data samples are used for training and validation, while the later data are used for testing. The later trials would leverage the knowledge or results obtained in the earlier trials. To make sure that there is no information leakage and fit the real clinical trial approval prediction setup, we leverage temporal split when partitioning the whole data into training/validation/testing datasets, following [8,11]. Specifically, as mentioned, all the trials we use in the learning process have both a start date and a completion date. We set up a split date to ensure that all the trials in the training and validation dataset are complete before the split date and all the trials in the test set are started after the split date [31]. For instance, in phase I, we trained the model on trials before 13 August 2014 and tested on trials post this date, as shown in Table 1.

### Evaluation

Our evaluation utilized various metrics, such as area under the precision–recall curve (PR-AUC), F1, area under the receiver operating characteristic curve (ROC-AUC), and accuracy. PR-AUC assesses the model’s ability to differentiate between positive and negative examples.

• PR-AUC. It quantifies the PR-AUC, representing how well the model separates positive and negative examples. PR-AUC focuses on the trade-off between precision (positive predictive value) and recall (true positive rate) across different probability thresholds.
• F1: The F1 score represents the harmonic mean of precision and recall. The F1 score is a single metric combining precision and recall into a single value to assess a classification model’s performance. The F1 score adeptly balances the trade-off between precision (the accuracy of positive predictions) and recall (the ability to identify all positive instances), ensuring a more accurate evaluation of the model’s accuracy.
• ROC-AUC: It focuses on the trade-off between true-positive rate (or recall) and false-positive rate across different probability thresholds. ROC-AUC quantifies the area under the ROC curve, which is a plot of true-positive rate against false-positive rate [32]. A higher ROC-AUC value indicates better model discrimination and the ability to distinguish between positive and negative examples.
• Accuracy: The ratio of correct predictions to the total number of samples.

We report the results of hypothesis testing in terms of *P* value to showcase the statistical significance of our method over the best baseline results. If the *P* value is smaller than 0.05, we claim that our method significantly outperforms HINT.

Furthermore, to promote transparency and facilitate reproducibility in the scientific community, we have made our code publicly available at the provided GitHub link (https://github.com/Vincent-1125/Uncertainty-Quantification-on-Clinical-Trial-Outcome-Prediction).

## Results

### Quantitative results

We only include HINT as the baseline model. The reason is that HINT outperforms a bunch of baseline methods (including traditional machine learning methods like logistic regression, random forest, AdaBoost and deep learning methods such as DeepEnroll, COMPOSE, etc.) across various phases statistically significantly [11].

We conduct experiments to evaluate the effect of using SC on trial approval prediction in the following aspects: We conducted a phase-level approval prediction to discern the enhancement offered by SC over the conventional model. Each trial phase was modeled individually using pretrained models from the HINT repository to ensure consistent and reproducible outcomes. We incorporated SC by setting a calibrated threshold *λ* on the training set. This threshold acted as a decision boundary to either retain or abstain from predictions based on the model’s confidence, as indicated by the softmax output.

**Fig. 4.**
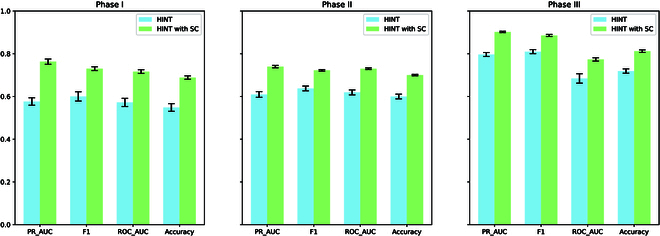
Phase-level approval prediction (average accuracy and SD). Our method significantly outperforms HINT (i.e., passing hypothesis testing, the *P* value is smaller than 0.05) in all the metrics in all the tasks.

The detailed results and observations are presented in Table 2 and Fig. 4. The results reveal that (a) we observed significant improvements across all phases, with phase I showing the most notable improvements. This indicates a strong adaptability of our model to early-stage trials. (b) All key performance metrics demonstrated marked improvements. The most striking gains are observed in PR-AUC. Although the F1 score’s enhancements were comparatively modest, they are indicative of a meaningful improvement in the model’s ability to maintain a balance between precision and recall—a critical consideration in the realm of imbalanced clinical trial datasets.

**Table 2.** Phase-level approval prediction. “*” means our method significantly outperforms HINT (i.e., passing hypothesis testing, the *P* value is smaller than 0.05.) in all the metrics in all the tasks.

Phase I			
	HINT	HINT with SC	Improvement
PR-AUC	0.5765 ± 0.0119	0.7631 ± 0.0119*	32.37%
F1	0.6003 ± 0.0091	0.7302 ± 0.0091*	21.64%
ROC-AUC	0.5723 ± 0.0084	0.7164 ± 0.0084*	25.18%
Accuracy	0.5486 ± 0.0046	0.6885 ± 0.0083*	25.50%
Retain rate	/	0.7874 ± 0.0267	/
Phase II
	HINT	HINT with SC	Improvement
PR-AUC	0.6093 ± 0.0131	0.7399 ± 0.0055*	21.43%
F1	0.6377 ± 0.0110	0.7224 ± 0.0036*	13.28%
ROC-AUC	0.6191 ± 0.0116	0.7299 ± 0.0038*	17.90%
Accuracy	0.5998 ± 0.0052	0.7002 ± 0.0031*	16.74%
Retain rate	/	0.5414 ± 0.0021	/
Phase III
	HINT	HINT with SC	Improvement
PR-AUC	0.7965 ± 0.0092	0.9022 ± 0.0031*	13.27%
F1	0.8098 ± 0.0093	0.8857 ± 0.0048*	9.37%
ROC-AUC	0.6843 ± 0.0220	0.7735 ± 0.0077*	13.04%
Accuracy	0.7190 ± 0.0063	0.8122 ± 0.0059*	18.69%
Retain rate	/	0.7117 ± 0.0172	/

The results indicate a consistent enhancement through the phases with SC. Phase I trials show a remarkable 32.37% increase in PR-AUC, indicating a substantial boost in the model’s precision and recall trade-off. Phases II and III also show notable improvements, albeit less pronounced than phase I. This could be due to the higher initial success rates in later phases, which leave less room for improvement. The data suggest that SC has the most significant impact where the uncertainty in predictions is greatest, thereby emphasizing the utility of SC in early-stage trials where risk assessment is critical.

**Fig. 5.**
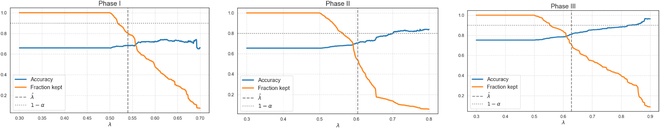
Trade-off between selective accuracy and fraction kept.

We also tune *λ* and show the change of selective accuracy and fraction kept (coverage) in Fig. 5. We find by increasing *λ*, accuracy would grow, and the fraction kept would decrease. That is to say, there is a trade-off between selective accuracy and fraction kept, as expected. We need to select appropriate *λ* to get a balance between them.

### Case study

**Fig. 6.**
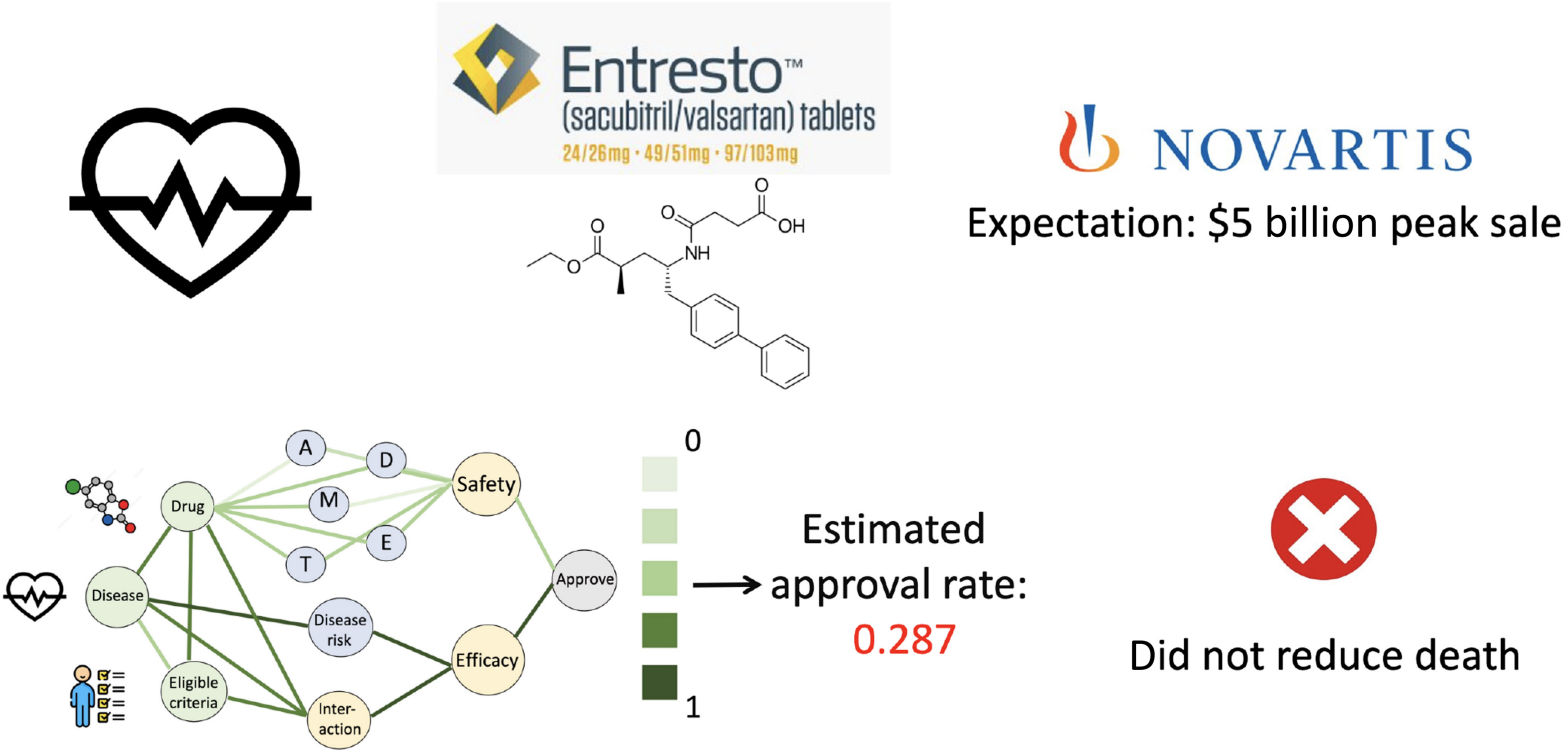
A case study for better interpretability. A phase III trial for heart failure was conducted across multiple countries and involved 4,822 patients. Our method estimates the trial’s chances of approval at 0.287, providing drug developers with confidence in the future prospects of the treatment. In addition, the heavier weighting in the lower components compared to the upper ones suggests that efficacy might be a key issue in the trial. This aligns with the actual outcomes observed. More details can be referred to Methods.

To show our method can predict clinical trial approval accurately and potentially save huge unnecessary costs in the case of failure, we demonstrate a representative example in Fig. 6. In 2019, Entresto emerged as a highly anticipated medication for heart failure, the principal cause of mortality in the United States. Backed by Novartis, Entresto was projected to reach peak sales of 5 billion dollars. Despite this, a comprehensive phase III trial conducted across multiple countries, involving 4,822 patients, yielded disappointing results. The drug failed to decrease mortality rates or achieve any other intended outcomes. Spanning from 2014 to 2019, this 5-year trial incurred an estimated cost of 200 million dollars (we estimate the cost by multiplying the median cost per patient by the total number of patients [33]). Next, we evaluate whether our approach can foresee such a failure in advance. By inputting the drug (Entresto), the condition (heart failure), and the eligibility criteria into our system, it forecasts a low probability of approval, at just 0.287. This suggests that our method might be capable of providing early warnings to healthcare professionals about the probable lack of success. We also visualize the weight of each edge in the interaction graph to interpret the results in Fig. 6. We find the weights in lower components are much darker than in upper components, revealing that efficacy may be the concern of the trial. This is consistent with the real results. This might serve as a prompt for clinical trial professionals to reassess the design of the trial and the characteristics of the drug.

### Results for disease groups

Our method’s effectiveness is assessed across various disease categories, such as cancer/neoplasm/tumor, chronic disease, pain, and cardiovascular disease, with the findings detailed in Table 3. It is noted that forecasts for cancer/neoplasm/tumor approvals are particularly challenging, exhibiting notably lower accuracy compared to other groups. In contrast, predictions for cardiovascular disease trials show the highest accuracy rate among all categories [34]. Trials related to pain and chronic diseases also demonstrate strong predictive performance.

**Table 3.** Results on different disease groups

Cohorts	% in test set	PR-AUC	F1	ROC-AUC
Neoplasm	13%	0.58 ± 0.01	0.56 ± 0.01	0.65 ± 0.02
Respiratory	9%	0.85 ± 0.02	0.87 ± 0.02	0.83 ± 0.01
Digestive	9%	0.80 ± 0.01	0.81 ± 0.01	0.87 ± 0.00
Nervous system	11%	0.68 ± 0.01	0.79 ± 0.01	0.79 ± 0.01

## Discussion

### Related work

#### Clinical Trial Approval Prediction

Publicly accessible data sources offer crucial insights for forecasting clinical trial approvals. The ClinicalTrials.gov database (publicly available at https://clinicaltrials.gov/), for example, lists 369,700 historical trials with significant details about them. Furthermore, the standard medical codes for diseases and their descriptions are available through the National Institutes of Health website (publicly available at https://clinicaltables.nlm.nih.gov/). The DrugBank database (publicly available at https://www.drugbank.ca/) provides biochemical profiles of numerous drugs, aiding in the computational modeling of these compounds.

In recent years, there have been various preliminary attempts to predict specific aspects of clinical trials, aiming to enhance prediction. These include using electroencephalographic measurements to gauge the impact of antidepressant therapies on alleviating depression symptoms [35], enhancing drug toxicity predictions through drug and target property characteristics [36,37], and leveraging phase II trial findings to forecast phase III trials results [38]. Recently, there is a growing inclination toward creating a universal strategy for predicting clinical trial approvals. As a preliminary effort, Andrew et al. [39] ventured beyond refining singular components, opting instead to forecast trial results for 15 ailment categories solely based on disease attributes through statistical analysis.

Notably, the work of Fu et al. [8,11] stands out in this field. Their contributions are 3-fold:

1. They established a formal modeling framework for clinical trial approval prediction, integrating information on drugs, diseases, and trial protocols.
2. By utilizing a comprehensive dataset from various online sources, including drug repositories, standardized disease codes, and clinical trial records, they have established a publicly available dataset TOP, based on which researchers can conduct general clinical trial approval prediction.
3. They developed HINT, a machine learning approach that explicitly models the components of clinical trials and constructs the intricate relationships among them. This method surpasses a range of traditional machine learning and deep learning models in performance.

#### Uncertainty Quantification

Regarding the prediction of clinical trial approvals, uncertainty quantification plays a pivotal role, as it aids in assessing the likelihood of trial success and informs decision-making processes. In this context, one principled framework that stands out is conformal prediction [40–42], a versatile and straightforward approach for generating prediction sets applicable to any model. In addition, SC, particularly suitable in binary classification scenarios, opts for abstaining from predictions when confidence is lacking. The idea of abstaining when the model is not certain originated in the last century [43,44]. More approaches were proposed in recent years, including using softmax probabilities [45], using dropout [46], and using deep ensembles [47]. Others incorporated abstention into model training [48–50] and learned to abstain on examples human experts were more likely to get correct [51–53]. On the theoretical level, early work characterized optimal abstention rules given well-specified models [54,55], with more recent work on learning with perfect precision [56,57] and guaranteed risk [45].

#### Interpretability of Deep Learning Model

With the widespread success of deep learning models across various fields, interpretability has increasingly garnered attention, particularly in the fields of medicine and healthcare, where it is crucial for decision-making and patient care [58]. There is a growing emphasis on the interpretability, transparency, and safety of processes, rather than only state-of-the-art achievements [59–62]. Interpretability refers to the ability to comprehend and articulate the functioning and decision-making process of the model. In assessing whether one model is more interpretable than another, we must consider these different dimensions of interpretability:

• Simplicity: Refers to the ease with which the model can be understood. Using simpler components is often regarded as more interpretable.
• Transparency: Involves understanding the internal mechanics of the model. Transparent models allow insight into how input data is transformed. This level of clarity is essential for meeting ethical and regulatory standards.
• Trustworthiness: Relates to the degree of reliability and confidence users have in the model’s predictions. This aspect is crucial for a model’s adoption in both academic and industrial areas.

For example, in the field of artificial intelligence (AI)-driven drug discovery, the interpretability of the model is important [63]. Regulatory bodies demand high transparency to understand how AI identifies potential drug candidates, ensuring safety and compliance in making critical R&D decisions.

### Discussion of results

The quantitative results reveal a consistent improvement across the stages with the application of SC. Specifically, phase I trials exhibit an impressive 32.37% uplift in PR-AUC, highlighting a significant enhancement in the model’s precision and recall balance. While phases II and III also demonstrate discernible advancements, these are not as marked as in phase I. This discrepancy may stem from the higher base success rates in the later stages, which naturally offer narrower margins for enhancement. The evidence points to SC having the most pronounced effect in scenarios with the highest prediction uncertainty, underlining SC’s value in early-phase trials where accurate risk evaluation is paramount.

### New findings

Our approach could potentially offer preliminary alerts to medical professionals regarding the likely failure of a treatment. In addition, by visualizing the significance attributed to each connection within the interaction graph, as illustrated in Fig. 6, we observe that the connections in the lower segments are significantly darker compared to those in the upper segments. This suggests that the trial’s effectiveness might be a point of concern, aligning with the actual outcomes observed. Such insights could prompt professionals involved in clinical trials to reevaluate the trial’s structure and the drug’s attributes.

### Contributions

The major contributions of this paper can be summarized as:

• Methodology: This paper introduces a novel approach that combines SC with the HINT, enhancing the model’s ability to withhold predictions in uncertain scenarios while also significantly improving the model’s interpretability. We provide a detailed explanation of the model’s operational mechanics and decision-making processes. Our model is built upon fundamental, trustworthy modules and uses a transparent modeling process throughout its formulation.
• Experimental results: Through comprehensive experiments, the paper demonstrates that this approach significantly improves performance metrics. Specifically, the proposed method achieved 32.37%, 21.43%, and 13.27% relative improvement in PR-AUC over the base model (HINT) in phase I, II, and III trial approval predictions, respectively. When predicting phase III, our method reaches 0.902 PR-AUC scores.
• Applications: The methodology presented has a specific focus on clinical trial approval predictions, highlighting its potential impact in this critical area of medical research.

### Practical applications

An AI-based clinical trial approvals prediction model can enhance practical clinical trials in the following 3 key areas:

• Enhanced cost predictions: A core feature of our model is its capability to generate more accurate cost predictions for clinical trials. It evaluates critical elements such as the length of the trial, participant count, and required resources to provide a precise financial forecast. This leads to improved budgeting and financial management.
• Reduced risk exposure: Our model plays a crucial role in pinpointing and reducing potential risks within clinical trials. Through detailed analysis, it flags potential issues such as drug inefficacy, safety hazards, or flaws in the trial design at an early stage, facilitating timely interventions.
• Improved trial selection: The model aids in selecting clinical trials with a higher probability of success by analyzing a range of factors. This not only ensures more efficient use of resources but also hastens the process of drug development.

### Limitations

We have to admit some limitations of the current work:

• Limited data and feature. There are a total of more than 340,000 clinical trials. However, the current dataset contains around 10,000 labeled trials. In addition, the current work utilizes disease, drug molecule, and trial protocol as the input feature of the trial and is unable to leverage other trial features, e.g., sponsor, country, etc.
• Poor prediction performance for some diseases. The prediction performance varies greatly from different disease subgroups. Because of the low frequency in the training data, AI models usually perform worse than average in rare disease-related trials, which are of high economic value to pharmaceutical companies. Thus, improving the prediction performance on rare diseases becomes a key challenge for clinical trial approval prediction models.

### Future work

Future work will focus on addressing the limitations of this work and can broaden the impacts of this work from the following 3 aspects:

• Larger dataset and richer feature. Currently, our clinical trial approval prediction dataset comprises thousands of trials with approval information for each phase. However, the total count of clinical trials exceeds 340,000 by the end of 2023. Expanding our dataset by incorporating additional data holds the potential to significantly benefit the approval study community. In addition, we plan to enrich the dataset by including more informative clinical trial features (e.g., sponsor and region) to further bolster the trial approval predictability.
• **Leveraging unlabeled data**. While hundreds of thousands of clinical trial records have been published, the approval status for the majority of them remains undisclosed. It is challenging and expensive to collect the approval information (serving as the label in the machine learning model) for the undisclosed ones. Nevertheless, leveraging unlabeled data has been widely demonstrated to be beneficial [64]. It is promising to investigate semi-supervised learning or zero-shot methods for leveraging those unlabeled clinical trial data and enhancing prediction performance.
• Fairness on various disease groups. The prediction performance across different disease groups varies greatly, e.g., with suboptimal results observed in rare disease-related trials. To ensure fairness in prediction across all disease groups, addressing the long-tailed issue is imperative. We plan to create digital twins or leverage transfer learning techniques to enhance the performance of rare disease-related trials.

### Conclusion

In conclusion, our study utilizing the HINT has presented a transformative approach in the domain of clinical trial approval prediction. By integrating the SC methodology for clinical trial approval predictions, we have not only addressed and quantified the inherent model uncertainty but also significantly enhanced the model’s interpretability, which has illustrated marked enhancements in performance.

The empirical results are compelling, demonstrating that SC confers a significant advantage, particularly evidenced by the pronounced improvements in PR-AUC across all phases of clinical trials. This is indicative of a more discerning model, capable of delivering higher precision in its predictions, especially in the critical early phases of clinical development.

The SC’s impact is most striking in phase I trials, where the model’s adaptability is crucial because of the higher uncertainty and variability. Despite the smaller gains in the F1 score, the consistent uplift across all metrics, including ROC-AUC and accuracy, underscores the overall increase in the model’s predictive reliability.

### Ethical Approval

This study, focusing on the development and application of AI for predicting clinical trial outcomes, was conducted in strict accordance with ethical principles and guidelines for research involving human data. This ethical approval confirms our commitment to conducting high-quality, ethical research that respects the rights and dignity of all individuals involved and contributes valuable insights to the field of AI in healthcare.

## Data Availability

All the data are publicly available at https://github.com/futianfan/clinical-trial-outcome-prediction. The code is publicly available at https://github.com/Vincent-1125/Uncertainty-Quantification-on-Clinical-Trial-Outcome-Prediction.
